# Volumetric analysis of bone resorption rate during lateral sinus lift: a retrospective study

**DOI:** 10.1186/s40729-026-00691-5

**Published:** 2026-05-13

**Authors:** Thouveny Gabriel, Cuny Constance, Zitouni Anissa, Pujos Théo, Canceill Thibault, Cousty Sarah, Dubuc Antoine

**Affiliations:** 1 Department of Oral Surgery and Implantology, Toulouse University Hospital, 46 Achille Viadieu Street, 31400 Toulouse, France; 2 InCOMM (Intestine ClinicOmics Microbiota and Metabolism, UMR1297 Inserm/Université de Toulouse, Institute of Metabolic and Cardiovascular Diseases (i2MC), Toulouse, France; 3 Departement of Oral Surgery, Limoges University Hospital, Limoges, France; 4 Centre d’Anthropobiologie et Génomique de Toulouse, Toulouse University, France; 5 Laboratoire Plasma et Conversion d’Energie - UMR 5213 – LAPLACE, Toulouse, France

**Keywords:** Sinus floor augmentation, Bone resorption, Maxillary sinus, Cone beam computed tomography, Schneiderian membrane

## Abstract

**Objective:**

To evaluate 1-year bone resorption after lateral sinus lift and assess the influence of contributing factors (Schneiderian membrane perforation, sinus anatomy, and surgical variables).

**Methods:**

This observational retrospective monocentric study compared CBCT-based volumetric outcomes in patients undergoing lateral sinus lift between 2020 and 2025. Scans were obtained immediately postoperatively and at 1-year follow-up, allowing precise three-dimensional assessment of grafted bone volume and evaluation of the impact of intraoperative membrane perforation. Bone volumes were measured using 3D Slicer^®^ with semi-automatic segmentation, a 3D imaging method enabling precise volumetric assessment of grafted bone. Multivariate analyses were performed to explore associations between anatomical features, graft characteristics, and bone resorption.

**Results:**

Forty-one sinuses were analyzed (mean age 62.8 ± 11.4 years). Bone loss was classified as < 10% (n = 4), 10–25% (n = 12), 25–50% (n = 18), and ≥ 50% (n = 7). No significant differences in age, sex, initial bone height, volume, or membrane thickness were observed between perforation and control groups. Multivariate analysis suggested that initial graft volume and sinus morphology influenced cases with ≥ 50% resorption, while properly managed membrane perforations did not significantly affect graft stability.

**Conclusions:**

One-year bone resorption after lateral sinus lift is multifactorial, and proper management of Schneiderian membrane perforations minimizes their impact on graft volume. The management of perforations and surgical technique appear to play a more significant role in graft stability than the simple occurrence of a perforation.

**What is known.**

Lateral sinus augmentation is a reliable procedure, yet significant graft resorption commonly occurs within the first postoperative year. Schneiderian membrane perforation is a frequent intraoperative event and is often presumed to negatively affect graft stability, although supporting volumetric evidence remains scarce.

**What this study adds.**

Based on standardized CBCT-derived volumetric measurements, this study demonstrates that adequately repaired membrane perforations do not compromise graft volume at 1 year. Instead, sinus anatomical configuration, particularly palato-vestibular angulation, together with initial graft volume, emerges as the primary driver of advanced resorption, underscoring the predominance of anatomical factors over perforation occurrence.

## Introduction

Alveolar bone loss in the posterior maxilla, often worsened by sinus pneumatization after tooth extraction, can complicate implant-based rehabilitation [1, 2]. Restoring sufficient bone height is therefore essential for predictable implant placement. The lateral sinus lift has become the gold standard technique, placing graft material between the sinus floor and the Schneiderian membrane to reconstruct the posterior maxilla and support implants [3, 4].

Despite high implant survival rates above 95–98% [5, 6], graft resorption remains a major limitation, particularly at the time of implant placement, as it can reduce bone volume and compromise primary stability. Reported volumetric losses range from 20 to 40% during the first postoperative year, mainly within the first six months [7, 8]. Understanding the factors that influence resorption is critical to optimize surgical planning and graft selection.

Resorption is influenced by graft type, with autogenous bone resorbing faster than allogeneic or xenogeneic substitutes [9, 10], and by surgical complications such as Schneiderian membrane perforations, which occur in around 30% of cases [11, 12]. Proper intraoperative management of perforations may help preserve graft volume [13, 14].

Preoperative sinus anatomy is a critical determinant of graft success in lateral window sinus augmentation [15]. CBCT evaluation of key anatomical parameters, including the presence and orientation of septa, septal angulation, edentulism pattern, Schneiderian membrane thickness, and lateral wall thickness, provides essential diagnostic information, as these factors can substantially influence graft behavior and overall clinical outcomes.

Evidence comparing graft resorption between lateral sinus lifts with and without intraoperative membrane perforations is limited. This retrospective cohort study aims to quantify volumetric bone resorption using CBCT and assess the impact of anatomical features and perforation management on graft stability. We hypothesize that when perforations are adequately repaired, sinus morphology contributes more to long-term resorption than the perforation itself.

## Material and methods

This retrospective monocentric cohort study was carried out at the Oral Surgery Unit of the Department of Dentistry, Toulouse University Hospital, and included all patients treated between 2020 and January 2025. The study protocol was approved by the university hospital ethics committee and registered in the internal registry of Toulouse University Hospital (RnIPH 2025—154) as well as in the national HDH registry (27,871,579). All data were anonymized prior to analysis.

This study follows the STROBE guidelines for reporting observational studies [16].

### Study population and patient selection

All adult patients (≥ 18 years) who underwent a lateral sinus lift between 2020 and 2025 in the Oral Surgery Department of Toulouse University Hospital were identified through the institutional database using the relevant billing codes (GBBA002 for unilateral procedures and GBBA364 for bilateral procedures). To minimize selection bias, these records were systematically cross-checked against the corresponding surgical reports. Exclusion criteria were duplicates, requirement for surgical revision with lateral approaches or crestal approaches, simultaneous implant placement, incomplete or poor-quality imaging, or absence of immediate post-operative CBCT. Eligible patients had no pre-existing sinus infection and complete immediate postoperative and 1-year CBCT scans.

**Fig. 1:**
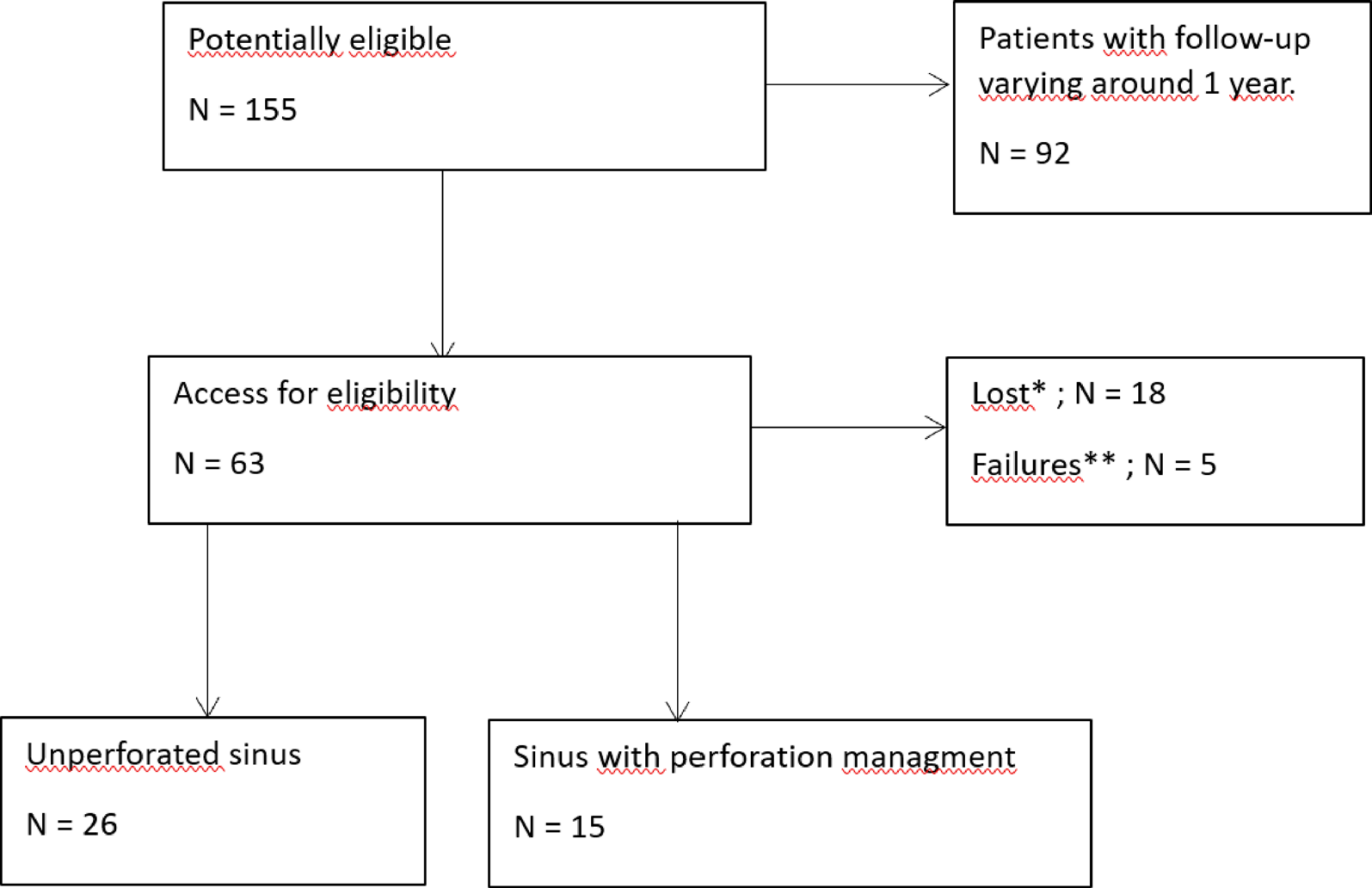
Flow diagram. *Follow-up outside the oral surgery department **Patients who underwent repeat surgery due to initial treatment failure were excluded

After filtering, 41 sinus lifts out of 153 were retained and classified into a “perforation group” (intraoperative Schneiderian membrane perforation) and a “control group” (no intraoperative complications). Flow chart is exposed in Fig. 1

### Diagnostic criteria and data collection measurement

**Fig. 2:**
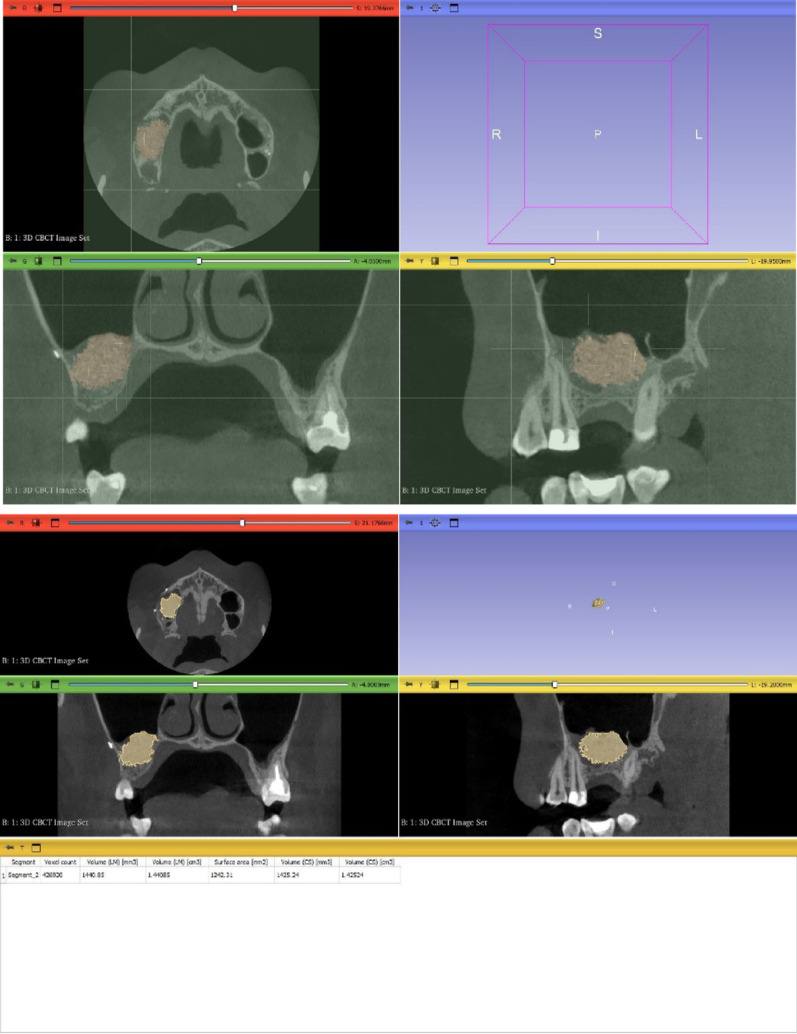
Workflow in 3D Slicer for sinus-lift volume segmentation segment creation **a** segmentation of densities **b** generation of the final 3D model

Two calibrated examiners independently conducted the preoperative assessment and volumetric analyses. Preoperative CBCT imaging was used to evaluate sinus anatomy and characterize exposure variables, including the presence or absence of septa at the operative site, septal angulation (< 30° or ≥ 30°), type of edentulism (bounded or non-bounded), Schneiderian membrane thickness (< 2 mm or ≥ 2 mm), and lateral bone wall thickness (< 5 mm or ≥ 5 mm). Intraoperative membrane perforations were identified visually and classified as small (< 5 mm) or large (≥ 5 mm). The graft material used was recorded from surgical reports and categorized as allogeneic (Biobank®) or xenogeneic (Bio-Oss®). Sinuses were considered healthy when no clinical or radiological signs of pathology were present. Clinical records, intraoperative observations, and CBCT data were used to collect all variables.Volumetric analysis was performed using 3D Slicer® (v4.11). Semi-automatic active-contour segmentation was carried out in the axial, sagittal, and coronal planes to obtain three-dimensional reconstructions. The region of interest (ROI) was strictly defined as the grafted bone volume. Segmentation was performed independently on two CBCT scans, immediate postoperative and 1-year follow-up, to allow longitudinal assessment of graft volume changes. Only the grafted region within the predefined ROI was included, while native cortical bone, sinus air cavity, and other non-grafted structures were systematically excluded to ensure accurate volume isolation. Discrepancies between the two examiners were resolved by consensus (GT/TP). Volumetric changes were calculated in cubic millimeters (mm^3^) (Fig. 2).

### Surgical procedure and perforation management

All procedures were performed by the same experienced surgeon using the standardized lateral window technique. Small perforations were repaired with monocryl 6.0 sutures, PRP or Pangen membranes, while large perforations were managed with the Loma Linda technique [17] which employs a resorbable collagen membrane to repair Schneiderian membrane perforations during sinus augmentation. Grafts consisted of allogenic or xenogenic bone, alone or in combination with guided bone regeneration.

### Bias and confounding control

Potential confounders such as age, sex, sinus anatomy, graft type, and surgical technique were recorded and adjusted for multivariate models. Selection bias was minimized through exhaustive sampling and manual case validation. Measurement bias was reduced by blinded independent examiners and standardized segmentation procedures.

### Sample size

The study size was determined by the number of eligible patients during the study period. With 41 sinus lifts, the study design allowed only limited sensitivity to detect small differences in final graft volume between groups (α = 0.05), particularly for small to moderate effect sizes.

### Statistical analysis

Quantitative variables were expressed as mean ± SD and compared using Student’s *t* test or Mann–Whitney U test, while categorical variables were analyzed with Fisher’s exact tests. For clinical relevance, perforation size was dichotomized as small (< 5 mm) or large (≥ 5 mm). Multivariate regression analyses adjusted for potential confounders, and sensitivity analyses compared healthy sinuses with those exhibiting perforations. The primary outcome was the volumetric change in grafted bone between immediate postoperative and 1-year CBCT scans, while secondary outcomes assessed the effects of perforation, sinus anatomy, graft material, and surgical technique on resorption. Statistical significance was set at *p* < 0.05 (two-tailed). Only patients with complete datasets were included. One-way ANOVA with Bonferroni post hoc tests was applied when normality and homogeneity of variance assumptions were met; otherwise, Kruskal–Wallis and Mann–Whitney U tests were used. Fisher’s exact test was used for qualitative variables.

## Results

Forty-one sinus lift with lateral approach were included (mean age 62.81 ± 11.43 years; 63.41% male [n = 26], 36.59% female [n = 15]). Bone loss categories were distributed as follows: < 10% (n = 4), 10–25% (n = 12), 25–50% (n = 18), and ≥ 50% (n = 7) (Table 1), based on the relative volumetric reduction between immediate and 1-year CBCT scans. These thresholds were defined to facilitate statistical comparison and reflect clinically relevant degrees of graft resorption.

**Table 1:** Comparison of patient and surgical characteristics according to bone loss categories

% Bone loss	< 10% *n* = 4	10 ≤ … < 25% *n* = 12	25 ≤ … < 50% *n* = 18	≥ 50% *n* = 7	*p*
Age	59.75 ± 17.54	63.42 ± 14.79	61.83 ± 9.53	66 ± 6.06	0.85
Sex (Female)	3 (75%)	3 (25%)	7 (38.89%)	2 (28.57%)	0.37
GBR*	4 (100%)	9 (75%)	14 (77.78%)	7 (100%)	0.44
Post operative Infection	2 (50%)	3 (25%)	2 (11.11%)	2 (28.57%)	0.27
Membrane perforation:					0.14
Small	1 (25%)	1 (8.33%)	5 (27.78%)	0 (0%)	
Large	0 (0%)	5 (41.67%)	1 (5.56%)	2 (28.57%)	
None	3 (75%)	6 (50%)	12 (66.67%)	5 (71.43%)	
Initial bone height (mm)	2.3 ± 1.39	3.48 ± 2.69	3.8 ± 2.33	3.3 ± 2.46	0.7
Initial volume (mm^3^)	1809 ± 1270.7	2529.5 ± 1270.6	2134.2 ± 906.5	2183.5 ± 1003.1	0.64
Final volume (mm^3^)	1743 ± 1271.8	2047.5 ± 994	1300.5 ± 660	969.2 ± 527.8	0.06
Septum present	1 (25%)	4 (33.33%)	4 (22.22%)	2 (28.57%)	0.95
Initial membrane thickness (mm)	1.55 ± 1.52	2.63 ± 2.98	3.83 ± 7.22	2.01 ± 1.36	0.81
Palatovestibular angle (°)	114.8 ± 37.8	107.7 ± 26.1	126.9 ± 18.8	105.1 ± 19.1	0.09
Embedded tooth loss	2 (50%)	3 (25%)	4 (22.22%)	0 (0%)	0.23

*Guided bone regeneration

Initial bone height (2.3 ± 1.4 to 3.8 ± 2.3 mm; *p* = 0.70), initial graft volume (1809 ± 1271 to 2529 ± 1271 mm^3^; *p* = 0.64), initial membrane thickness (1.6 ± 1.5 to 3.8 ± 7.2 mm; *p* = 0.81), and palato-vestibular angle (105.1° ± 19.1 to 126.9° ± 18.8; *p* = 0.09) were comparable across groups. No significant differences were observed among bone loss groups regarding demographic variables, postoperative infection, or the presence of sinus septa (all p-values are greater than 0.05). The frequency of Schneiderian membrane perforation was comparable between groups (*p* = 0.14). Small perforations, managed intraoperatively with platelet-rich fibrin (PRF) or simple suturing, were observed in all bone-loss categories except the ≥ 50% group, while large perforations, managed with the Loma Linda technique, were slightly more frequent in cases with ≥ 50% bone loss.

Final graft volume showed a consistent decrease with increasing bone loss categories (from 2047.5 ± 994 mm^3^ in the 10–25% group to 969.2 ± 528 mm^3^ in the ≥ 50% group), without reaching statistical significance (*p* = 0.06). Although not statistically confirmed, this trend is clinically coherent, reflecting the expected relationship between higher resorption rates and reduced graft volume. Although the overall ANOVA did not reach statistical significance (p = 0.06), post hoc comparisons were performed as an exploratory analysis. A difference was observed between the 10–25% and ≥ 50% resorption groups (*p* < 0.05, uncorrected), suggesting a possible effect that may reflect limited statistical power rather than a definitive intergroup difference.

### Principal component analysis

**Fig. 3:**
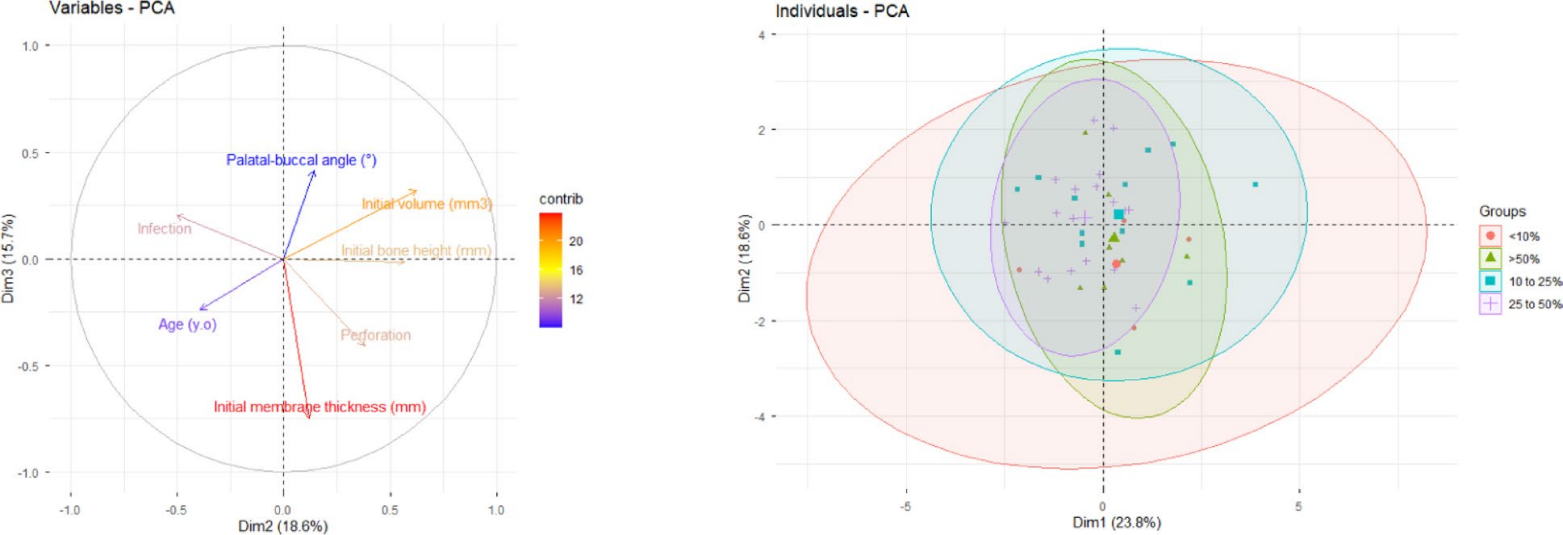
PCA of patient and surgical variables by bone loss category. **a** Variable contributions to PCs (vectors colored by relative weight). **b** Patients on PC1 versus PC2, colored/shaped by bone loss (< 10%, 10–25%, 25–50%, > 50%); ellipses indicate within-group variance. Axes show % variance explained. Highlights associations between anatomical/surgical factors and bone resorption

Principal component analysis (PCA) identified three main components (PC1, PC2, and PC3) explaining most of the total variance among measured variables (respectively 23.8, 18.6 and 15.7%) (Fig. 3).

PC1 was primarily associated with initial graft volume and palato-vestibular angle, which strongly influenced cases exhibiting ≥ 50% bone loss.

PC2 was mainly driven by initial membrane thickness and minimal residual bone height, but showed no distinct clustering pattern among bone loss categories.

PC3 was characterized by contributions from initial bone volume and palato-vestibular angle, explaining the variability observed within the 10–25% and ≥ 50% resorption groups.

Overall, the PCA highlights the multifactorial nature of post-sinus lift bone resorption, with sinus morphology, particularly the palato-vestibular configuration and initial graft volume, emerging as potential contributors to higher resorption rates, independent of membrane perforation occurrence.

These multivariate findings prompted further evaluation of the clinical relevance of each parameter in relation to the existing literature, as detailed in the following discussion section.

## Discussion

### Main findings

This retrospective cohort study evaluated volumetric bone resorption 1 year after 41 lateral sinus lift and investigated whether intraoperative Schneiderian membrane perforations influenced graft stability. The results demonstrated that, although membrane perforation occurred in approximately one-third of cases, it did not significantly affect the final graft volume when adequately managed using sutures, platelet-rich fibrin (PRF), or the Loma Linda technique [18].

Although our ANOVA did not demonstrate a statistically significant effect of graft volume and sinus morphology on resorption (*p* = 0.06), the observed trend is clinically plausible and aligns with prior studies [19]. This suggests that larger graft volumes and more open sinus angles may predispose to increased volumetric loss.. These findings suggest that while demographic and anatomic parameters did not differ substantially, greater bone loss was associated with lower final graft volume and potentially influenced by initial sinus morphology.

To investigate relationships between anatomical and volumetric factors, a principal component analysis (PCA) was performed. This approach revealed that initial graft volume and sinus morphology, particularly the palato-vestibular angle, were key contributors to bone resorption patterns. While membrane perforations alone did not compromise graft stability, larger graft volumes combined with wide sinus configurations were associated with greater volumetric loss, highlighting the interplay between surgical and anatomical factors in determining long-term outcomes.

Collectively, these results support the notion that successful sinus augmentation depends less on the mere presence of membrane perforation than on its proper management and the patient’s sinus anatomy. These findings add quantitative evidence into the multifactorial determinants of graft resorption after sinus lift surgery. While previous studies have primarily focused on implant survival rates or general complication frequencies, few have quantitatively assessed how membrane perforation and sinus anatomy interact to influence postoperative volumetric outcomes.

The present study therefore contributes to filling this gap by combining CBCT-based three-dimensional volumetric assessment with multivariate analysis. This approach enables a more comprehensive understanding of the factors that govern graft stability and allows direct comparison with previous reports on perforation management and sinus morphology, as discussed below.

### Originality and comparison with existing literature

The present study adds an innovative contribution by providing a detailed three-dimensional assessment based on highly precise, standardized CBCT-derived volumetric measurements, allowing a more accurate evaluation of the true impact of membrane perforations on graft remodeling. Most of the available literature has focused on implant survival or complication rates rather than on the direct volumetric consequences of perforations [11, 13, 14, 20–23].

The present data underscore the central role of sinus morphology and initial graft configuration in shaping long-term volumetric stability. In line with the structure revealed by our principal component analysis, PC1, driven predominantly by initial graft volume and the palato-vestibular angle, captured most of the variance associated with cases exhibiting ≥ 50% bone loss. These parameters reached their highest values in this group, with initial graft volumes up to 2529 ± 1271 mm^3^ and palato-vestibular angles approaching 126.9° ± 18.8, indicating a biomechanically less favorable sinus environment. These findings directly echo previous studies. [24] reported that sinus wall angulation critically affects graft integration and bone gain, while [25, 26] emphasized the influence of lateral wall configuration and membrane characteristics on postoperative stability.

PC2, primarily capturing membrane thickness and minimal residual bone height, did not distinguish the bone-loss categories, a pattern consistent with the substantial variability of these measurements in our sample (e.g., membrane thickness 1.6 ± 1.5 to 3.8 ± 7.2 mm, residual bone height 2.3 ± 1.4 to 3.8 ± 2.3 mm). This lack of discriminatory power aligns with literature suggesting that, although these parameters contribute to the risk of perforation, they exert a weaker influence on long-term volumetric preservation(25, 26]. PC3, combining contributions from the initial bone volume and palato-vestibular angle, accounted for the intra-group variability particularly evident in the 10–25% and ≥ 50% resorption categories. The observed decline in final graft volume, from 2047.5 ± 994 mm^3^ in the 10–25% group to 969.2 ± 528 mm^3^ in the ≥ 50% group, further supports the view that sinus anatomy interacts with the initial graft configuration to modulate secondary resorption, consistent with the biomechanical interpretation proposed by [25].

In our series, the frequency of Schneiderian membrane perforations was comparable across bone-loss categories, although large perforations were slightly more frequent in cases with ≥ 50% resorption. All large perforations were managed using the Loma Linda technique, employing a resorbable collagen membrane to seal the defect and stabilize the graft. [27] demonstrated reliable graft containment and predictable healing, while [28, 29] confirmed its effectiveness for both small and large perforations. More recent developments, pouch-type or modified Loma Linda techniques [17], further enhance graft stability and reduce migration. Collectively, these findings support our results, showing that even in cases with large perforations, management with the Loma Linda technique ensures consistent postoperative outcomes.

Our results suggest that small Schneiderian membrane perforations, when managed intraoperatively with PRF or simple suturing, do not significantly influence postoperative bone resorption. [30] reported that appropriate management of minor membrane perforations preserves graft stability and implant success.

Taken together, the convergence of our PCA-based analysis, the quantitative CBCT-derived anatomy, and the established evidence on membrane repair techniques highlights the multifactorial nature of volumetric stability in sinus augmentation. Our results indicate that wider palato-vestibular angles and larger initial graft volumes are the primary determinants of volumetric reduction, largely independent of membrane perforation, whose incidence did not differ significantly between groups (*p* = 0.14). This reinforces the importance of comprehensive preoperative anatomical assessment, careful management of intraoperative complications, and individualized surgical planning to optimize graft longevity.

To better understand the clinical implications of these associations, it is essential to interpret the observed trends in light of sinus physiology, graft remodeling dynamics, and the surgical management of the Schneiderian membrane, as discussed in the following section.

### Interpretation and clinical relevance

Post-sinus lift bone resorption is multifactorial, reflecting the interplay of anatomical, material-related, and technical factors. While graft type or membrane thickness were not individually significant, PCA highlighted their combined influence with variables such as initial graft volume and sinus wall angles, particularly in patients with major resorption. Consistent with [24, 26] sinus morphology, including residual bone height, wall configuration, and septa, plays a key role in surgical outcomes, while thicker Schneiderian membranes (> 2 mm) increase technical difficulty and risk of perforation [31]. Collectively, these findings underscore that careful preoperative assessment and tailored surgical planning are essential to optimize volumetric stability after sinus lift procedures.

### Strengths and limitations

The study has some limitations.

A primary limitation of this study is the high exclusion rate, with only 41 out of 153 initial sinus lifts included in the final volumetric analysis. This substantial reduction was primarily due to our strict inclusion criteria requiring high-quality CBCT scans both immediately postoperatively and at the 1-year follow-up. In routine clinical practice in our department, an immediate control CBCT is not systematically prescribed unless explicitly required, leading to a large number of missing follow-up scans. Furthermore, patient relocation, financial considerations, or refusal of additional radiation exposure contributed to the loss to follow-up. This high exclusion rate may introduce a selection bias, as the analyzed cohort represents patients who strictly adhered to the follow-up protocol and required comprehensive imaging. Consequently, this could potentially affect the generalizability of our findings, though the demographic and initial anatomical characteristics of our final sample remain representative of standard sinus lift populations.

The relatively small sample size and limited statistical power may have constrained the ability to detect subtle but clinically relevant associations, study also presents several notable strengths. The main strength of this work lies in directly comparing bone resorption between sinus lifts with and without Schneiderian membrane perforation, an area still underexplored in the literature. All procedures were performed by experienced clinicians following a standardized protocol, minimizing procedural variability. Volumetric outcomes were assessed using a validated CBCT segmentation method, ensuring objective and reproducible measurements. Finally, principal component analysis provided a nuanced understanding of the complex interplay between anatomical, procedural, and material-related factors.

### Clinical implications

Proper intraoperative management of membrane perforation may minimize its impact on graft resorption. This challenges the assumption that perforations automatically indicate poor prognosis. These findings may influence clinical decision-making regarding grafting strategies and timing of implant placement.

Further prospective studies with larger cohorts and comprehensive clinical data, including smoking status, metabolic conditions, and systemic health variables, are needed to confirm and extend these results.

## Conclusion

This study demonstrates that volumetric bone resorption after lateral sinus lift is influenced by a complex interplay of anatomical, material-related, and technical factors. Importantly, intraoperative Schneiderian membrane perforations, whether small, managed with PRF or suturing, or large, repaired using the Loma Linda technique, did not significantly compromise graft stability when properly managed. Instead, wider palato-vestibular angles and larger initial graft volumes emerged as the primary determinants of volumetric loss, underscoring the critical role of sinus morphology and graft configuration in postoperative outcomes. These findings highlight the importance of thorough preoperative anatomical assessment and individualized surgical planning to optimize graft longevity. While the study’s limited sample size and retrospective design warrant cautious interpretation, it provides valuable quantitative evidence on the multifactorial determinants of graft resorption, reinforcing the need for larger prospective studies to further clarify these relationships and guide clinical decision-making.

## Data Availability

The datasets generated and/or analyzed during the current study are available from the corresponding author on reasonable request.
